# Durable Response to Pembrolizumab With Chemotherapy in EGFR-Mutated Squamous Cell Lung Cancer: A Case Report

**DOI:** 10.7759/cureus.81973

**Published:** 2025-04-09

**Authors:** Taira Ninomaru, Sho Yoshimura, Satoshi Mukaida, Kenjiro Matsuo, Yohei Kimura, Tokiko Nakai

**Affiliations:** 1 Respiratory Medicine, Hyogo Prefectural Harima-Himeji General Medical Center, Himeji, JPN; 2 Pathology, Hyogo Prefectural Harima-Himeji General Medical Center, Himeji, JPN

**Keywords:** egfr-tki, epidermal growth factor receptor gene mutation, non-small cell lung cancer, pembrolizumab, squamous cell lung cancer

## Abstract

Epidermal growth factor receptor (EGFR)-tyrosine kinase inhibitor (TKI) is the standard treatment for *EGFR*-mutated non-small cell lung cancer (NSCLC), although a small subset of patients does not respond to EGFR-TKI, especially in patients with a smoking history and squamous cell histology. Additionally, even given an initial good response, acquired resistance to EGFR-TKI is inevitable. More individualized treatment could be necessary in certain types of *EGFR*-mutated NSCLC. We herein report a case of *EGFR*-mutated metastatic lung squamous cell carcinoma with a high tumor proportion score (TPS) of programmed death ligand 1 (PD-L1), where chemotherapy plus pembrolizumab therapy had a durable efficacy. In this case, chemotherapy plus pembrolizumab therapy was administered, though EGFR-TKI is the standard first-line therapy of *EGFR*-mutated NSCLC. Three years after treatment initiation, no sign of recurrence was observed. This case suggests that a detailed evaluation of a patient's background is important in making an effective personalized therapeutic strategy.

## Introduction

Epidermal growth factor receptor (*EGFR*) mutation is the most frequently found oncogene driver mutation in non-small cell lung cancer (NSCLC), but its prevalence in lung squamous cell carcinoma (LSCC) is rare [1]. Although EGFR-tyrosine kinase inhibitors (TKI) are the most preferred treatment option for *EGFR*-mutated NSCLC, some research revealed comparatively poor efficacy of EGFR-TKI for *EGFR*-mutated LSCC [2,3]. This evidence has been only extracted from subset data or small sample-sized studies, and no large-scale prospective trial has been obtained due to its rarity of population. Though EGFR-TKI is chosen as the first-line therapy for *EGFR*-mutated LSCC, acquired resistance inevitably follows despite its initial remarkable response [4]. On the contrary, immune checkpoint inhibitor (ICI) is known for its durable response in certain populations [5]. ICI therapy exhibits limited activity in most *EGFR*-mutated NSCLC, even in high programmed death ligand 1 (PD-L1) population [6]. However, some *EGFR*-mutated NSCLC patients do benefit from ICI, and durable response is obtained [7]. A predictive marker for ICI in *EGFR*-mutated NSCLC would be beneficial in making the best therapeutic strategy. We herein report a case of *EGFR*-mutated LSCC that responded durably to pembrolizumab.

## Case presentation

**Figure 1:**
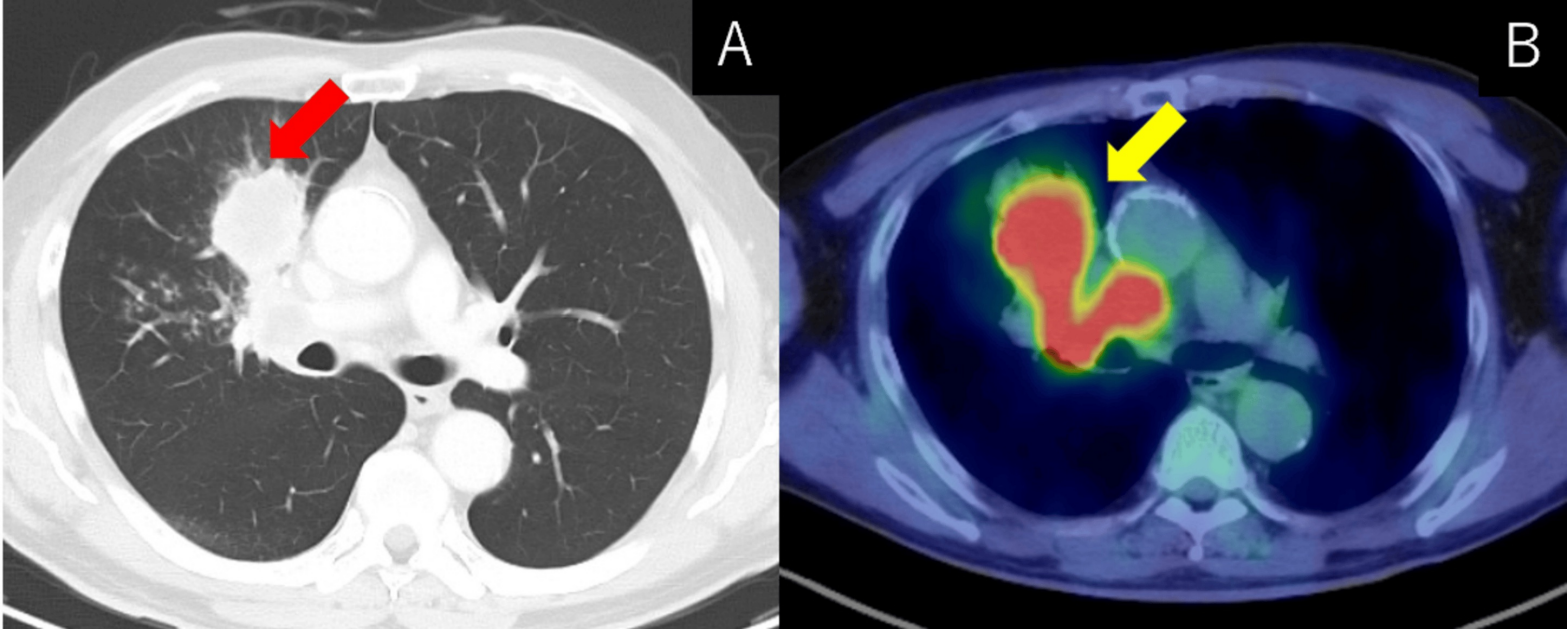
Radiological finding before treatment (A) Computed tomography (CT) revealed a mass in the right middle lobe and enlarged mediastinum lymph node (red arrow). (B) Positron-emission tomography (PET)-CT revealed increased uptake in the right middle lobe and mediastinum lymph node (yellow arrow).

A 65-year-old man with a current smoking history presented to our hospital with an abnormal finding on a chest X-ray that was detected during a regular medical check-up. He was asymptomatic and had no prior medical history. His Eastern Cooperative Oncology Group (ECOG) performance status was 0. No abnormality was revealed on physical examination. Chest computed tomography (CT) revealed a mass in the right middle lobe, mediastinal lymph node enlargement, and small nodules in bilateral lungs. Positron emission tomography (PET)-CT revealed increased uptake in the right middle lobe and mediastinum lymph node (Figure 1).

**Figure 2:**
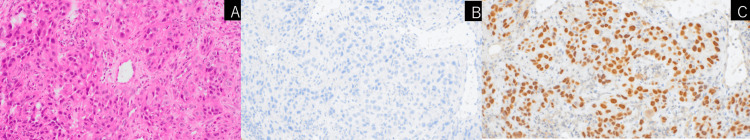
Pathological images Histology of specimen from right middle lobe stained with (A) Hematoxylin-eosin staining, (B) Thyroid transcription factor-1 (TTF-1), and (C) P40 staining by immunohistochemistry.

Brain magnetic resonance imaging (MRI) showed no brain metastasis. We performed bronchoscopy from the primary lesion. Pathological analysis revealed no apparent keratinizing tendency along with thyroid transcription factor 1 (TTF-1) negative and p40 positive by immunohistochemistry (Figure 2).

**Figure 3:**
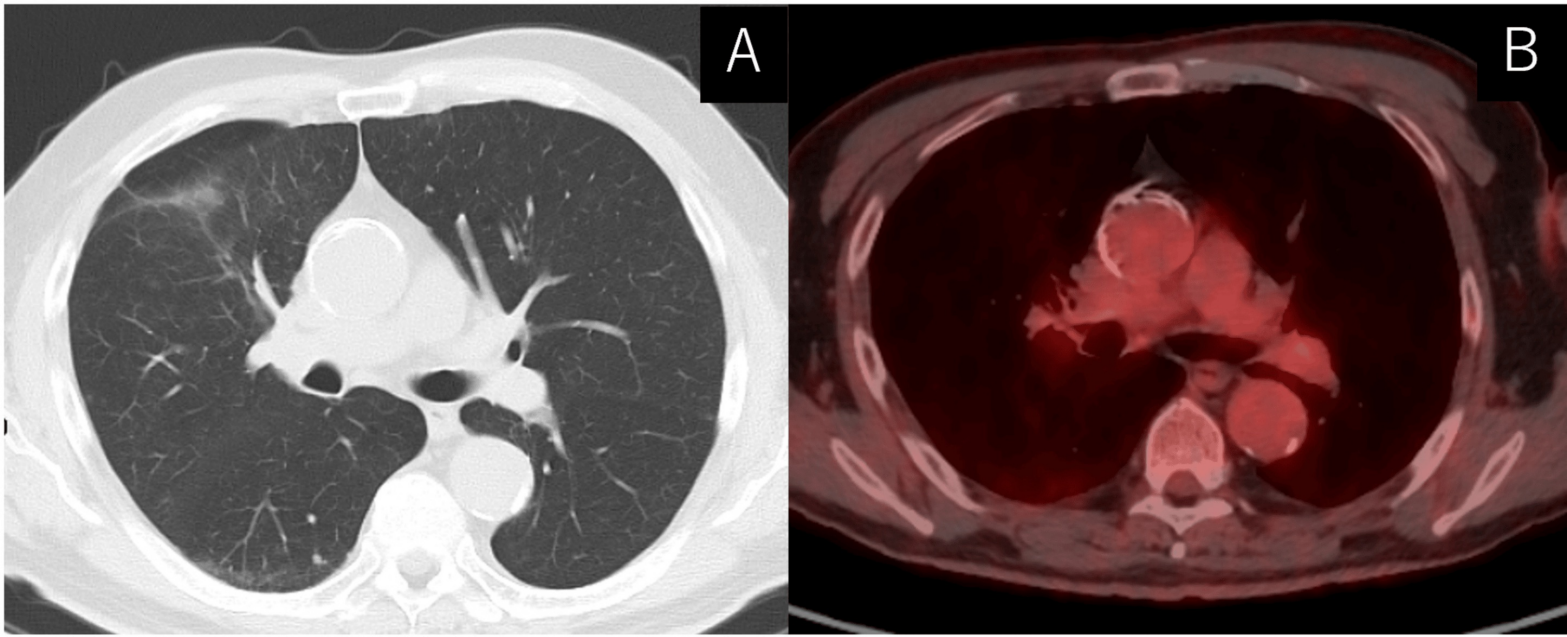
Radiological image after treatment (A) Computed tomography (CT) revealed shrinkage of mass and lymph node three years after treatment initiation. (B) Positron-emission tomography (PET)-CT revealed no uptake neither in shrunk mass or mediastinum.

With pathological findings, we diagnosed metastatic LSCC (cT3N2M1c: cStage IVB). The EGFR polymerase chain reaction (PCR) test revealed exon 19 deletion. The tumor proportion score (TPS) of PD-L1 was 95%. Though EGFR-TKI is the standard first line for *EGFR*-mutated NSCLC, the patient preferred to receive chemotherapy plus immunotherapy. We started to administer carboplatin plus nab-paclitaxel plus pembrolizumab. After four cycles of chemotherapy and immunotherapy, CT revealed shrinkage of the primary tumor and metastatic lesions, and no adverse event was observed. We then proceeded to pembrolizumab monotherapy. After switching to pembrolizumab monotherapy, primary tumor and metastatic lesions continued to shrink. All through two years after treatment initiation, a good response had been consistently observed without any adverse event. We completed the scheduled two-year treatment and moved to regular hospital visitation and serological and radiological observation. Three years after treatment initiation, PET/CT revealed no sign of recurrence (Figure 3).

## Discussion

EGFR-TKIs were reported to be less effective in *EGFR*-mutated LSCC than in adenocarcinoma (ADC), although no large-scale prospective trial has been conducted [2,3]. The disease control rate is significantly lower, and progression-free survival and overall survival are significantly shorter in LSCC than in ADC [2]. However, some *EGFR*-mutated LSCC obtain clinical benefit from EGFR-TKI, and it is necessary to distinguish those who are likely to obtain benefit from those who are not.

As *EGFR*-mutated LSCC harboring ADC propensity was reported to respond well to EGFR-TKI, it could be an ideal first-line treatment for *EGFR*-mutated LSCC with the following clinical and pathological factors: female, never-smoker, high carcinoembryonic antigen (CEA) level, and TTF-1 positive [8]. As for *EGFR*-mutated LSCC with the clinical and pathological factors of male, current smoker, high CK19 fragment, and p63 positive, first-line EGFR-TKI therapy may not be effective [8]. Chemotherapy or chemotherapy plus ICI could be an option for this patient subset, as in our case.

In general, ICI efficacy to *EGFR*-mutated NSCLC remains controversial. Even PD-L1 could not precisely predict ICI efficacy in *EGFR*-mutated NSCLC [6]. This is due to the fact that tumor-infiltrating lymphocytes (TILs), as well as PD-L1 on the surface of tumor cells, play an important role in suppressing tumor cells with ICI [9]. In order to predict ICI efficacy more precisely, information on both PD-L1 and TIL is necessary, but information on the presence of TIL is not easily available in clinical practice. Tumor mutation burden (TMB), which is associated with high levels of neoantigens, is also an indicator of ICI efficacy in NSCLC [10]. In *EGFR*-mutated NSCLC, TMB was low compared to *EGFR*-wild NSCLC [11], which could be a reason for ICI inefficacy. On the other hand, TMB is associated with smoking, and its association is dose-dependent [12]. Additionally, though not significantly different, TMB tends to be higher in *EGFR*-mutated LSCC than in *EGFR*-mutated ADC [13]. Considering smoking status and histology, our case was expected to have high TMB [14]. Given that TMB is negatively associated with clinical outcomes of *EGFR*-mutated NSCLC, EGFR-TKI therapy could have been ineffective in our case. 

## Conclusions

*EGFR*-mutated NSCLC is a heterogenous population, which could respond differently to ICI or EGFR-TKI depending on its clinical, pathological and molecular factors. In light of this, we should make a personalized therapeutic strategy based on each patient’s background, including gender, smoking history, tumor marker, and pathological findings. 

## References

[1] Miyamae Y, Shimizu K, Hirato J (2011). Significance of epidermal growth factor receptor gene mutations in squamous cell lung carcinoma. Oncol Rep.

[2] Liu Y, Zhang Y, Zhang L (2017). Efficacy of epidermal growth factor receptor-tyrosine kinase inhibitors for lung squamous carcinomas harboring EGFR mutation: a multicenter study and pooled analysis of published reports. Oncotarget.

[3] Fang W, Zhang J, Liang W (2013). Efficacy of epidermal growth factor receptor-tyrosine kinase inhibitors for Chinese patients with squamous cell carcinoma of lung harboring EGFR mutation. J Thorac Dis.

[4] Kobayashi S, Boggon TJ, Dayaram T (2005). EGFR mutation and resistance of non-small-cell lung cancer to gefitinib. N Engl J Med.

[5] Reck M, Rodríguez-Abreu D, Robinson AG (2016). Pembrolizumab versus chemotherapy for PD-L1-positive non-small-cell lung cancer. N Engl J Med.

[6] Toki MI, Mani N, Smithy JW (2018). Immune marker profiling and programmed death ligand 1 expression across NSCLC mutations. J Thorac Oncol.

[7] Nogami N, Barlesi F, Socinski MA (2022). IMpower150 final exploratory analyses for atezolizumab plus bevacizumab and chemotherapy in key NSCLC patient subgroups with EGFR mutations or metastases in the liver or brain. J Thorac Oncol.

[8] Hata A, Katakami N, Yoshioka H (2014). How sensitive are epidermal growth factor receptor-tyrosine kinase inhibitors for squamous cell carcinoma of the lung harboring EGFR gene-sensitive mutations?. J Thorac Oncol.

[9] Teng MW, Ngiow SF, Ribas A, Smyth MJ (2015). Classifying cancers based on T-cell infiltration and PD-L1. Cancer Res.

[10] Rizvi NA, Hellmann MD, Snyder A (2015). Cancer immunology. Mutational landscape determines sensitivity to PD-1 blockade in non-small cell lung cancer. Science.

[11] Dong ZY, Zhang JT, Liu SY (2017). EGFR mutation correlates with uninflamed phenotype and weak immunogenicity, causing impaired response to PD-1 blockade in non-small cell lung cancer. Oncoimmunology.

[12] Wang X, Ricciuti B, Nguyen T (2021). Association between smoking history and tumor mutation burden in advanced non-small cell lung cancer. Cancer Res.

[13] Jin R, Peng L, Shou J (2021). EGFR-mutated squamous cell lung cancer and its association with outcomes. Front Oncol.

[14] Offin M, Rizvi H, Tenet M (2019). Tumor mutation burden and efficacy of EGFR-tyrosine kinase inhibitors in patients with EGFR-mutant lung cancers. Clin Cancer Res.

